# Recovery Profiles of Sevoflurane and Desflurane with or without M-Entropy Guidance in Obese Patients: A Randomized Controlled Trial

**DOI:** 10.3390/jcm11010162

**Published:** 2021-12-29

**Authors:** Yu-Ming Wu, Yen-Hao Su, Shih-Yu Huang, Po-Han Lo, Jui-Tai Chen, Hung-Chi Chang, Yun-Ling Yang, Yih-Giun Cherng, Hsiang-Ling Wu, Ying-Hsuan Tai

**Affiliations:** 1 Department of Anesthesiology, Shuang Ho Hospital, Taipei Medical University, New Taipei City 23561, Taiwan; 15538@s.tmu.edu.tw (Y.-M.W.); 10689@s.tmu.edu.tw (S.-Y.H.); 12006@s.tmu.edu.tw (P.-H.L.); 19240@s.tmu.edu.tw (J.-T.C.); 09331@s.tmu.edu.tw (H.-C.C.); 10500@s.tmu.edu.tw (Y.-L.Y.); stainless@s.tmu.edu.tw (Y.-G.C.); 2 Department of Anesthesiology, School of Medicine, College of Medicine, Taipei Medical University, Taipei 11031, Taiwan; 3 Division of General Surgery, Department of Surgery, Shuang Ho Hospital, Taipei Medical University, New Taipei City 23561, Taiwan; su11066@tmu.edu.tw; 4 Department of Surgery, School of Medicine, College of Medicine, Taipei Medical University, Taipei 11031, Taiwan; 5 Department of Anesthesiology, Taipei Veterans General Hospital, Taipei 11217, Taiwan; hlwu9@vghtpe.gov.tw; 6 School of Medicine, National Yang Ming Chiao Tung University, Taipei 11221, Taiwan

**Keywords:** bariatric surgery, depth of anesthesia, electroencephalographic monitoring, emergence agitation, morbid obesity

## Abstract

Obesity increases the risk of prolonged emergence from general anesthesia due to the delayed release of anesthetic agents from body fat. This trial aimed to evaluate the effects of sevoflurane and desflurane along with anesthetic depth monitoring on emergence time from anesthesia in obese patients. Adults with a body mass index ≥ 30 kg·m^−2^ undergoing laparoscopic sleeve gastrectomy at a medical center were randomized into four groups: sevoflurane or desflurane anesthesia with or without M-Entropy guidance on anesthetic depth in a ratio of 1:1:1:1. In the M-Entropy guidance groups, the dosage of sevoflurane and desflurane was adjusted to achieve response and state entropy values between 40 and 60 during surgery. In the non-M-Entropy guidance groups, the dosage of anesthetics was titrated according to clinical signs. Primary outcome was time to spontaneous eye opening. A total of 80 participants were randomized. Compared to sevoflurane, desflurane anesthesia significantly reduced the time to spontaneous eye opening [mean difference (MD): −129 s; 95% confidence interval (CI): −211, −46], obeying commands (−160; −243, −77), tracheal extubation (−172; −266, −78), and leaving operating room (−148; −243, −54). M-Entropy guidance further reduced time to eye opening (MD: −142 s; 99.2% CI: −276, −8), tracheal extubation (−199; −379, −19), and leaving operating room (−190; −358, −23) in the desflurane but not the sevoflurane group. M-Entropy guidance significantly reduced the risk of agitation during emergence, i.e., risk difference: −0.275 (95% CI: −0.464, −0.086); and number needed to treat: 4. Compared to sevoflurane, using desflurane to maintain general anesthesia accelerated the return of consciousness in obese patients. M-Entropy guidance further hastened awakening in patients using desflurane and prevented emergence agitation.

## 1. Introduction

Obesity is a growing epidemic, affecting about 650 million adults worldwide in 2016 [1]. The global prevalence of obesity has almost tripled in the past four decades, exerting a heavy burden on healthcare system [1,2]. Obesity substantially increases the risks of metabolic, cardiovascular, and respiratory diseases, as well as several types of cancer [3]. The global volume of surgery for obese patients is forecast to increase as a result of the growing prevalence of, and diseases related to, obesity [4].

Recovery from general anesthesia may be compromised in obese patients due to the delayed release of lipid-soluble anesthetic agents from excessive adipose tissue [5]. In addition, obese patients are susceptible to the respiratory depression effects of anesthetics, which potentiates the development of respiratory adverse events (e.g., airway obstruction and hypoxemia) after surgery [6]. The latest Enhanced Recovery After Surgery (ERAS) guidelines do not recommend specific anesthetic regimens for early emergence from general anesthesia in bariatric surgery due to conflicting results in the current literature [7,8,9,10,11,12,13,14,15,16]. Some studies reported that desflurane has a consistent and rapid recovery profile in the obese population compared to sevoflurane, isoflurane and propofol [8,9,10,11,12,13]. However, other investigators demonstrated similar awakening times between sevoflurane and desflurane anesthesia [14,15,16]. Overall, the current evidence is insufficient to determine the optimal anesthetic agent for obese patients in terms of immediate recovery from general anesthesia.

Electroencephalography (EEG) neuromonitoring is effective in guiding an optimal range of anesthetic depth during general anesthesia [17]. A meta-analysis showed that bispectral index (BIS)-guided anesthesia enhances emergence from general anesthesia in nonobese patients compared to clinical signs [17]. However, the effect of anesthetic depth monitoring on emergence from anesthesia remains largely unexplored in obese patients [18,19]. Furthermore, there are few studies examining the interplay between different anesthetic agents and EEG neuromonitoring and their joint effect on anesthetic emergence in obese patients.

We conducted a prospective, four-arm, randomized controlled trial to investigate the effects of desflurane versus sevoflurane, together with spectral entropy monitoring, for anesthetic depth on emergence time from general anesthesia in obese patients. Specifically, we hypothesized that using desflurane for maintenance of anesthesia along with spectral entropy monitoring reduce the time to emergence from anesthesia in obese patients undergoing bariatric surgery.

## 2. Materials and Methods

This trial obtained the approval from the Joint Institutional Review Board of Taipei Medical University in Taiwan (TMU-JIRB-N202002076). It was registered in an international directory, www.clinicaltrials.gov (accessed on 27 September 2021) (identifier: NCT04395248). Informed verbal and written consent were obtained from all participants before randomization. This study was performed in accordance with the Helsinki Declaration and relevant regulations.

### 2.1. Patient Selection Criteria

**Figure 1:**
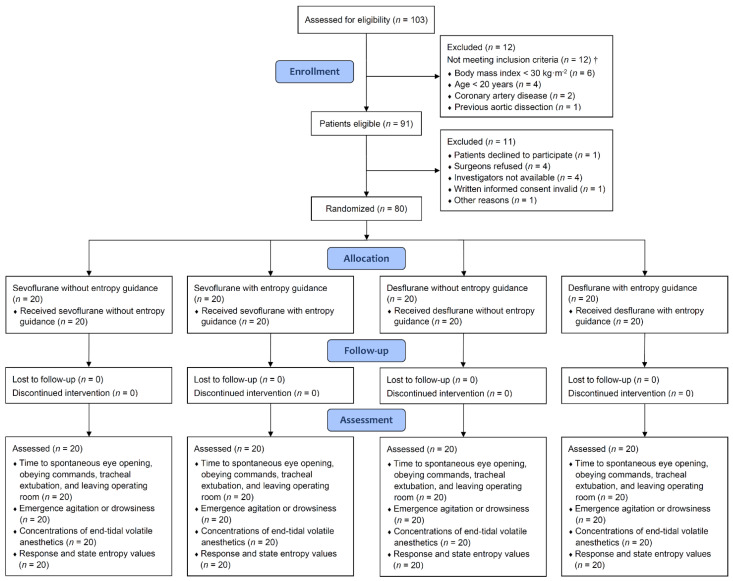
Consolidated Standards of Reporting Trials flow diagram. † Not mutually exclusive, since patients could have more than one exclusion criterion.

We conducted a four-arm parallel randomized controlled trial to prospectively recruit patients undergoing laparoscopic sleeve gastrectomy at a medical center between May 2020 and August 2021. Inclusion criteria were age 20 to 65 years and body mass index equal to or greater than 30 kg·m^−2^. Exclusion criteria were use of hypnotics or antipsychotics within 30 days before surgery, known cerebrovascular disease, stage 4 or 5 chronic kidney disease (estimated glomerular filtration rate < 30 mL·min·1.73 m^−2^), significant cardiovascular disease (e.g., coronary artery disease and previous aortic dissection), peripheral capillary oxygen saturation < 90% in room air, pregnant women, and patient refusal (Figure 1). All operations were performed by the same team of surgeons, using the same surgical techniques.

### 2.2. Randomization Methods

Patients were randomly allocated into four groups (sevoflurane without M-Entropy guidance, sevoflurane with M-Entropy guidance, desflurane without M-Entropy guidance, and desflurane with M-Entropy guidance) in a ratio of 1:1:1:1. The RAND function of Statistics Analysis System (SAS), version 9.4 (SAS Institute Inc., Cary, NC, USA) was used to produce a sequence of random permuted blocks of four. After obtaining informed consent, each patient was given a unique identifier and a group assignment by the principal investigator. The assignments were then enclosed in envelopes and sealed. An independent attending anesthesiologist (Y.-M.W. or S.-Y.H.) opened the relevant envelope upon the patient’s arrival at the operating room and administered the assigned intervention.

### 2.3. Anesthesia Management and M-Entropy Guidance

In the operating room, a M-Entropy™ sensor and a S/5™ module (GE Healthcare, Helsinki, Finland) were applied to all patients’ foreheads before induction of anesthesia, according to the manufacturer’s recommendations. This was connected to a M-Entropy monitor that was concealed from the patient and surgeons. General anesthesia was induced with propofol at a dose of 1.5–2.0 mg·kg^−1^ body weight and fentanyl 2–3 μg·kg^−1^ total body weight. An infusion of rocuronium 0.8–1.0 mg·kg^−1^ body weight was administered to facilitate endotracheal intubation. After intubation, pressure-controlled ventilation with a peak pressure < 30 cm H_2_O and a positive end expiratory pressure of 5 cm H_2_O was applied to achieve a tidal volume 8–10 mL·kg^−1^ ideal body weight and a respiratory rate of 10–15 min^−1^, as well as to maintain end-tidal carbon dioxide below 45 mm Hg during surgery. The inspiratory oxygen fraction was set between 0.6 and 0.8 to maintain peripheral capillary oxygen saturation above 95%. Anesthesia was maintained using volatile anesthetics of sevoflurane or desflurane with a fresh gas flow of 6 L·min^−1^ during the first 5 min, and 1.5–2 L·min^−1^ thereafter. The vaporizer was set at 2 vol% for sevoflurane and 6 vol% for desflurane during the first 5 min.

In the M-Entropy guidance groups, the dosage of sevoflurane and desflurane was adjusted to achieve response and state entropy values between 40 and 60. In the non-M-Entropy guidance groups, the dosage of anesthetics was titrated according to clinical signs and judgement. Typically, this was to maintain a mean arterial pressure within a 20% range of the baseline and a heart rate within the range 50 to 100 beats·min^−1^. In case of signs of inadequate anesthesia (e.g., movement, cough, and swallowing), the anesthetic dosage was increased. M-Entropy monitoring was continued in the non-M-Entropy groups, but the entropy values were concealed from the anesthetist in charge. Entropy values, hemodynamic, and expiratory gas data were recorded in 5-min intervals. At the end of wound closure, volatile anesthetics were discontinued, and the fresh gas flow was returned to 6 L·min^−1^ with 100% oxygen. Once the train-of-four count recovered to 1–4, sugammadex dosed at 2 mg·kg^−1^ ideal body weight +40% was administered to reverse neuromuscular blockade [20]. Manual-breathing support was then used to maintain end-tidal carbon dioxide below 45 mm Hg until the return of spontaneous ventilation.

### 2.4. Outcome Measurement

Primary outcome was time to spontaneous eye opening, defined as the interval between cessation of volatile anesthetics and patient’s eye opening. Secondary outcomes included time to obeying verbal command (sustained head lift or handgrip for 5 s), tracheal extubation, and leaving operating room, as well as events of agitation or drowsiness during emergence. The Richmond Agitation-Sedation Scale (RASS) was used to evaluate the levels of agitation and sedation [21]. Agitation was defined as a RASS score +2 to +4, and drowsiness as −2 to −5. Patients and surgeons were blinded to group allocations. In addition, the anesthetic and M-Entropy monitors were concealed from an independent nurse anesthetist (Y.-L.Y.), who was blinded to group allocations. This anesthetist determined the time to recovery from anesthesia and measured the levels of agitation and sedation during emergence.

### 2.5. Sample Size Estimation

According to a prior study, at least 36 patients in each group of sevoflurane or desflurane are needed to detect a difference of 186 s of time to spontaneous eye opening, accepting a type I error of 5% and type II error of 20%, with a mean anticipated time to eye opening of 450 s and a standard deviation of 200 s in the sevoflurane group [22,23]. We enrolled 40 subjects in each group to compensate for possible dropouts.

### 2.6. Statistical Analysis

Shapiro-Wilk tests and Kolmogorov-Smirnov tests were used to examine the normality of included variables. Normally distributed variables were summarized using mean ± standard deviation. Non-normally distributed data were presented as medians with interquartile range, minimum, and maximum. The distributions of baseline patient characteristics and outcome variables were compared across four groups using ANOVA or Kruskal-Wallis tests for continuous variables, as appropriate. Chi-square tests or Fisher’s exact tests were used to compare categorical variables across four groups, as appropriate. For pairwise comparisons, either *t* tests or Mann-Whitney U tests were used for continuous variables, and chi-square tests or Fisher’s exact tests for categorical variables. We considered *p* < 0.05 to be statistically significant for a two-sided test. A Bonferroni correction to the significance criterion was applied for multiple pairwise comparisons. All the statistical analyses were conducted using SAS software.

## 3. Results

### 3.1. Baseline Patient and Clinical Characteristics

Table 1 shows the baseline characteristics of the enrolled patients. The distributions of demographics, body mass index, American Society of Anesthesiologists physical status, lifestyle factors, and coexisting diseases were generally balanced across the four groups. Regarding preoperative laboratory data, there was a significant difference in the estimated glomerular filtration rate across the four groups, but pairwise comparisons did not show any differences among groups (data not shown). There was no difference in the baseline response and state entropy values, doses of intravenous anesthetics, duration of anesthesia, or amount of intravenous fluids among the four groups, either (Table 2).

### 3.2. Intraoperative Hemodynamic Changes

The heart rate, mean arterial pressure, body temperature, and peripheral capillary oxygen saturation among the four groups were generally comparable before and after induction of anesthesia, during pneumoperitoneum, at the cessation of volatile anesthetics, and after tracheal extubation (Supplementary Table S1). There was a difference in the body temperature at 5 min after start of pneumoperitoneum across groups, but pairwise comparisons showed no difference among groups (data not shown).

### 3.3. Study Outcomes

Compared to sevoflurane, desflurane anesthesia significantly reduced time to spontaneous eye opening [mean difference (MD): −129 s, 95% confidence interval (CI): −211, −46], obeying commands (MD: −160 s, 95% CI: −243, −77), tracheal extubation (MD: −172 s, 95% CI: −266, −78), and leaving operating room (MD: −148 s, 95% CI: −243, −54). In addition, desflurane was associated with lower average values of response and state entropy and higher time percentages of response, as well as state entropy values < 40 compared, to sevoflurane (Table 3). There was no difference in the rate of emergence agitation between sevoflurane and desflurane.

Overall, M-entropy guidance did not affect times to spontaneous eye opening, obeying commands, tracheal extubation, or leaving operating room (Table 3). However, M-Entropy guidance significantly decreased time to spontaneous eye opening (MD: −142 s, 99.2% CI: −276, −8), tracheal extubation (MD: −199 s, 99.2% CI: −379, −19), and leaving operating room (MD: −190 s, 99.2% CI: −358, −23) in patients receiving desflurane anesthesia (Table 4 and Table 5). In addition, M-Entropy guidance reduced the risk of emergence agitation, with a risk difference of −0.275 (95% CI: −0.464, −0.086) and a number needed to treat of 4 (Table 3). M-Entropy guidance was associated with higher time percentages of response and state entropy values ranging from 40 to 60. The average response and state entropy values of M-Entropy guidance groups were significantly higher than those without M-Entropy guidance. In addition, M-Entropy guidance significantly reduced the average age-adjusted minimum alveolar concentration of end-tidal desflurane, but not sevoflurane, during surgery.

## 4. Discussion

In this randomized clinical trial, desflurane significantly accelerated emergence from anesthesia compared to sevoflurane in obese patients undergoing bariatric surgery. In addition, M-Entropy guidance for anesthetic depth further shortened the awakening time in patients receiving desflurane but not sevoflurane. Our results also suggested that M-Entropy guidance was effective in reducing the development of emergence agitation. These benefits were related to a shallower anesthetic depth, as demonstrated by the higher response and state entropy values in M-Entropy guidance groups. Our findings indicated that desflurane with M-Entropy guidance may serve as a practicable strategy for early and uneventful emergence from anesthesia in obese surgical patients.

Obese patients are prone to delayed emergence from general anesthesia and to the lasting respiratory depression effects of anesthetic agents [5,6]. However, there is still a lack of consensus regarding the optimal protocol to accelerate recovery from anesthesia in the obese population [7]. Clinical trials have reported that BIS-guided desflurane anesthesia not only achieve faster eye opening, airway reflex recovery, tracheal extubation, and orientation, but also increase modified Aldrete scores and oxygen saturations in postanesthesia care units compared to sevoflurane, isoflurane, and propofol [8,9,10,11,12,13]. However, other investigators refuted the advantages of desflurane over sevoflurane in immediate recovery [14,15,16]. These discrepancies might result from the heterogeneity in patients’ body mass indexes, administered adjuvant hypnotic drugs, anesthetic depth targets, and types of surgery [8,9,10,11,12,13,14,15,16]. Elbakry and colleagues recently showed that total intravenous anesthesia using propofol and dexmedetomidine was effective in reducing postoperative pain intensity, analgesic consumptions, and length of postanesthesia care unit stay among morbidly obese patients compared to desflurane anesthesia [24]. The time to achieve an Aldrete score of 10 was similar between the two methods, but emergence times and agitation were not assessed [24]. Given that the current pharmacokinetic models (e.g., Marsh and Schnider) may not be accurate in obese patients [25], more studies are needed to evaluate the optimal infusion regimens and clinical benefits of total intravenous anesthesia among obese patients in the context of enhanced recovery after surgery.

Few studies have investigated the benefits of EEG guidance of anesthetic depth in the immediate recovery from anesthesia in obese patients. In a randomized controlled trial, Ibraheim and colleagues reported that obese patients receiving BIS-guided sevoflurane anesthesia had significantly faster awakening and shorter extubation times, and lower sevoflurane consumption and medical costs compared to those without BIS monitoring [19]. However, this study was limited by a small patient sample and no evaluation of other anesthetics [19]. It is noteworthy that few studies have simultaneously investigated the impact of different anesthetic agents and EEG guidance on patients’ wake-up times. The present study showed that patients receiving desflurane combined with M-Entropy guidance had more rapid recovery compared to those with sevoflurane or without M-Entropy guidance. These results provide clinical insights to prevent delayed emergence from anesthesia following surgery for obese patients.

We propose the following possible mechanisms for the inconsistent effects of M-Entropy guidance on emergence time between sevoflurane and desflurane. First, awareness of faster wash-out and wake-up from desflurane compared to sevoflurane might contribute to the use of high concentrations of desflurane in the absence of anesthetic depth neuromonitoring. [8,9,10,11,12,13]. This was reflected by the higher end-tidal concentration of desflurane but not sevoflurane in patients without M-Entropy guidance. Second, De Baerdemaeker and colleagues showed that desflurane was associated with better hemodynamic controllability and lower risk of hypotension in obese patients compared to sevoflurane [9]. The anesthetists in the desflurane groups were perhaps less concerned about hypotension, which might have given rise to deeper anesthesia associated with desflurane when anesthesia was not guided by M-Entropy. Third, M-Entropy guidance increased the intraoperative time percentages of response entropy and state entropy ranging from 40 to 60 by 31.9% and 30.1% in desflurane, but only by 14.5% and 17.7% in sevoflurane. The difference in hypnotic controllability between sevoflurane and desflurane might explain our results.

Interestingly, our study demonstrated a potential benefit of M-Entropy guidance in reducing emergence agitation in obese adults following bariatric surgery. Studies have shown that obesity and inhalational anesthesia (especially anesthetics with low blood-gas solubility, such as sevoflurane and desflurane) are risk factors for agitation and delirium during emergence from anesthesia in adults [26,27]. However, to date, few studies have investigated the potential effect of EGG monitoring and emergence agitation in adults [28]. In children, studies have shown that neither deep hypnosis (BIS value < 45) nor prolonged emergence affects the risk of emergence agitation [29,30]. Moreover, we did not observe a significant difference in the risk of emergence agitation between sevoflurane and desflurane, in contrast with other studies [31]. Our findings warrant future studies to evaluate the effects of shallow or deep anesthesia, anesthetic depth guidance, and different anesthetic agents on the development of emergence agitation in obese patients.

This study had some limitations. First, the number of participants in the trial was modest, which may have given rise to some underpowered statistics in our intergroup comparisons. Second, the administration of volatile anesthetics was primarily based on clinical judgement and was not standardized by protocols. Consequently, the anesthetists’ preferences for different anesthesia practices might affect the generalizability of the study results. Third, we did not evaluate the use of volatile anesthetics and the medical cost of general anesthesia, which precluded a cost-benefit analysis of the different interventions applied in this study. Fourth, the anesthetists who titrated the volatile anesthetics according to entropy values or clinical signs during surgery could not be blinded, which might have biased the study results. Fifth, we did not evaluate total intravenous anesthesia, which may be protective against emergence agitation compared to inhalational anesthesia [32,33]. Sixth, the differences in times to emergence were approximately 2 to 5 min among the groups in our study; this possibly represents a low clinical significance in daily practice. Finally, the use of desflurane has been phased out in many countries in view of its impact on global warming [34]. Nevertheless, our results suggested that M-Entropy guidance can reduce the time percentage of deep anesthesia and the risk of emergence agitation in sevoflurane anesthesia. This finding has clinical implications in the anesthesia care of patients with obesity.

## 5. Conclusions

The use of desflurane to maintain general anesthesia significantly shortened the time to emergence from anesthesia in obese patients compared to sevoflurane. Additionally, the utilization of M-Entropy neuromonitoring to guide intraoperative depth of anesthesia further reduced the recovery time in patients receiving desflurane rather than sevoflurane. M-Entropy guidance might be effective in preventing the occurrence of emergence agitation. These findings provide evidence to facilitate the postoperative recovery from anesthesia and to decrease complications associated with delayed emergence and emergence agitation in obese patients undergoing surgery.

## Figures and Tables

**Table 1:** Baseline patient characteristics.

	SEVO without M-Entropy *n* = 20		SEVO with M-Entropy *n* = 20		DES without M-Entropy *n* = 20		DES with M-Entropy *n* = 20		*p*
Age, years	38.0	7.3	37.9	10.7	37.0	10.5	33.9	7.5	0.4471
Sex, male	12	60.0	7	35.0	11	55.0	8	40.0	0.3328
Body mass index, linear, kg·m^−2^	41.4	37.5, 46.4 (32.3, 56.3)	38.3	34.4, 41.8 (31.9, 52.5)	41.2	37.2, 47.5 (32.7, 59.2)	39.3	34.4, 42.2 (30.9, 47.9)	0.2512
Body mass index, binary, kg·m^−2^									0.4660
<40	8	40.0	12	60.0	8	40.0	11	55.0	
≥40	12	60.0	8	40.0	12	60.0	9	45.0	
Waist circumference, cm	126.0	12.6	125.4	14.1	127.0	17.5	122.6	12.9	0.7919
ASA physical status									0.4660
II	8	40.0	12	60.0	8	40.0	11	55.0	
III	12	60.0	8	40.0	12	60.0	9	45.0	
Current cigarette smoking	7	35.0	6	30.0	11	55.0	10	50.0	0.3236
Current alcohol drinking	4	20.0	4	20.0	4	20.0	2	10.0	0.8177
Coexisting disease									
Hypertension	11	55.0	5	25.0	7	35.0	4	20.0	0.0925
Diabetes mellitus	3	15.0	3	15.0	4	20.0	4	20.0	>0.9999
Obstructive sleep apnea	8	40.0	9	45.0	9	45.0	6	30.0	0.7410
Fatty liver	17	85.0	15	75.0	15	75.0	17	85.0	0.7949
Preoperative blood test									
Hemoglobin, g·dL^−1^	14.7	13.9, 15.5 (12.3, 17.8)	14.3	13.2, 15.1 (8.7, 16.8)	14.8	14.0, 15.8 (12.5, 17.7)	14.5	13.9, 15.1 (10.8, 16.6)	0.4802
eGFR, mL·min·1.73 m^−2^	93.5	84.4, 116.5 (53.9, 166.7)	119.9	103.1, 129.3 (82.8, 153.9)	99.2	87.0, 126.8 (70.4, 166.5)	121.9	99.8, 133.2 (80.5, 189.9)	0.0105
Sodium, mmol·L^−1^	139	137, 140 (136, 144)	139	137, 141 (130, 145)	139	137, 140 (135, 144)	138	137, 139 (134, 143)	0.8009
Potassium, mmol·L^−1^	3.9	3.8, 4.0 (3.3, 4.4)	3.9	3.6, 4.1 (3.4, 4.4)	3.8	3.7, 4.0 (3.5, 4.4)	4.1	3.8, 4.1 (3.3, 4.2)	0.4356
Alanine aminotransferase, U·L^−1^	30	27, 40 (18, 84)	34	25, 44 (12, 159)	28	24, 35 (15, 80)	36	22, 62 (15, 242)	0.5160
Aspartate aminotransferase, U·L^−1^	34	21, 44 (17, 77)	37	23, 58 (16, 142)	33	22, 38 (18, 67)	40	23, 69 (12, 305)	0.4865

Values are mean with standard deviation, counts with percent, or median with interquartile range (minimum and maximum). Abbreviations: ASA, American Society of Anesthesiologists; DES, desflurane; eGFR, estimated glomerular filtration rate; SEVO, sevoflurane.

**Table 2:** Baseline entropy values and intraoperative anesthetic parameters.

	SEVO without M-Entropy *n* = 20		SEVO with M-Entropy *n* = 20		DES without M-Entropy *n* = 20		DES with M-Entropy *n* = 20		*p*
RE value before induction	98	97, 99 (93, 100)	98	97, 98 (91, 98)	98	97, 99 (95, 100)	98	97, 99 (89, 100)	0.6215
SE value before induction	88	87, 90 (86, 94)	88	86, 89 (82, 90)	89	88, 89 (82, 90)	87	86, 89 (72, 91)	0.0896
Intravenous anesthetics									
Lidocaine, mg	100	80, 100 (60, 100)	80	80, 100 (60, 100)	95	80, 100 (80, 100)	80	80, 100 (60, 100)	0.3515
Fentanyl, μg	200	150, 200 (150, 250)	175	150, 200 (100, 200)	200	150, 200 (150, 250)	200	188, 200 (150, 250)	0.1270
Propofol, mg	200	155, 200 (150, 200)	160	145, 200 (130, 200)	200	150, 200 (120, 200)	175	150, 200 (120, 200)	0.1486
Dexamethasone, mg	5	5, 5 (5, 5)	5	5, 5 (5, 5)	5	5, 5 (5, 5)	5	5, 5 (5, 5)	>0.9999
Glycopyrrolate, mg	0.2	0.2, 0.2 (0.2, 0.2)	0.2	0.2, 0.2 (0.2, 0.2)	0.2	0.2, 0.2 (0.2, 0.2)	0.2	0.2, 0.2 (0.2, 0.2)	>0.9999
Rocuronium, mg	100	75, 115 (60, 140)	95	80, 100 (60, 160)	100	88, 123 (70, 140)	95	85, 135 (70, 200)	0.2216
Sugammadex, mg	185	150, 200 (135, 220)	165	155, 193 (150, 200)	200	170, 200 (130, 210)	170	150, 200 (130, 220)	0.2877
Duration of anesthesia, min	120	110, 148 (75, 280)	116	100, 135 (90, 210)	123	100, 150 (90, 170)	128	91, 146 (65, 200)	0.8408
Amount of intravenous fluids, mL	800	625, 1000 (350, 1350)	750	650, 900 (350, 1200)	700	600, 850 (500, 1000)	725	600, 800 (400, 1000)	0.5477

Values are median with interquartile range (minimum and maximum). Abbreviations: DES, desflurane; RE, response entropy; SE: state entropy; SEVO, sevoflurane.

**Table 3:** Study outcomes of desflurane versus sevoflurane and M-Entropy guidance versus no guidance.

	Sevoflurane Anesthesia *n* = 40		Desflurane Anesthesia *n* = 40		*p*	No M-Entropy Guidance *n* = 40		M-Entropy Guidance *n* = 40		*p*
Time to spontaneous eye opening, s	454	257, 569 (149, 982)	315	182, 392 (121, 778)	0.0034	372	249, 569 (125, 982)	333	193, 475 (121, 814)	0.0922
Time to obeying commands, s	494	391, 609 (210, 1158)	361	233, 434 (130, 825)	<0.0001	422	330, 609 (180, 1158)	384	273, 520 (130, 884)	0.1060
Time to tracheal extubation, s	571	450, 697 (232, 1066)	385	254, 490 (160, 1144)	0.0001	504	388, 664 (205, 1144)	412	281, 590 (160, 1047)	0.0675
Time to leaving operating room, s	849	677, 911 (443, 1400)	683	538, 800 (410, 1255)	0.0008	765	665, 902 (443, 1372)	707	549, 855 (410, 1400)	0.1658
Emergence agitation	13	32.5	10	25.0	0.4586	17	42.5	6	15.0	0.0066
Drowsiness after tracheal extubation	3	7.5	2	5.0	>0.9999	2	5.0	3	7.5	>0.9999
Intraoperative awareness or recall	0	0	0	0	NA	0	0	0	0	NA
Time percentage of RE > 60	24.4	13.3, 45.1 (1.9, 77.3)	12.0	7.2, 18.8 (2.5, 51.5)	0.0004	15.5	8.3, 31.1 (1.9, 77.3)	18.3	11.0, 28.9 (2.5, 66.7)	0.5475
Time percentage of RE ranged 40–60	61.6	48.8, 77.3 (22.2, 92.5)	72.2	48.7, 83.3 (9.7, 95.0)	0.2346	51.7	30.2, 67.8 (9.7, 90.7)	78.9	68.4, 83.3 (27.8, 95.0)	<0.0001
Time percentage of RE < 40	3.8	0, 13.2 (0, 55.6)	8.3	0, 33.2 (0, 87.1)	0.0589	17.4	1.4, 42.2 (0, 87.1)	0	0, 5.9 (0, 19.2)	<0.0001
Average RE value	56	51, 63 (42, 70)	52	46, 55 (36, 62)	0.0002	50	44, 58 (36, 70)	55	52, 59 (47, 68)	0.0045
Time percentage of SE > 60	21.1	13.0, 37.9 (1.9, 77.3)	11.0	6.0, 16.7 (2.5, 51.5)	0.0010	12.8	7.1, 31.1 (1.9, 77.3)	16.0	11.0, 24.2 (2.5, 61.1)	0.4883
Time percentage of SE ranged 40–60	67.4	46.9, 80.0 (22.2, 92.5)	71.0	51.3, 81.3 (9.7, 95.0)	0.8663	50.0	34.1, 69.6 (9.7, 88.9)	77.8	71.8, 84.0 (33.3, 95.0)	<0.0001
Time percentage of SE < 40	3.8	0, 13.8 (0, 55.6)	14.3	3.4, 34.9 (0, 87.1)	0.0074	19.7	4.0, 49.1 (0, 87.1)	3.4	0, 9.6 (0, 28.6)	<0.0001
Average SE value	55	49, 60 (41, 68)	50	45, 53 (35, 60)	0.0003	48	43, 56 (35, 68)	54	50, 57 (45, 66)	0.0063
Average level of end-tidal SEVO, %	1.63	1.49, 1.76 (1.21, 2.29)	NA	NA	NA	1.66	1.57, 1.76 (1.21, 2.16)	1.54	1.46, 1.83 (1.33, 2.29)	0.4091
Average level of end-tidal SEVO, aaMAC	0.76	0.71, 0.83 (0.57, 1.12)	NA	NA	NA	0.78	0.73, 0.83 (0.57, 0.99)	0.73	0.69, 0.84 (0.63, 1.12)	0.4165
Average level of end-tidal DES, %	NA	NA	4.69	4.26, 5.20 (2.83, 6.83)	NA	4.85	4.60, 5.23 (3.65, 6.83)	4.41	4.03, 5.09 (2.83, 5.95)	0.0531
Average level of end-tidal DES, aaMAC	NA	NA	0.69	0.62, 0.78 (0.42, 0.95)	NA	0.73	0.67, 0.80 (0.51, 0.95)	0.64	0.59, 0.75 (0.42, 0.85)	0.0358

Values are counts with percent or median with interquartile range (minimum and maximum). Abbreviation: aaMAC, age-adjusted minimum alveolar concentration; DES, desflurane; RE, response entropy, SE: state entropy; SEVO, sevoflurane; NA, not applicable.

**Table 4:** Study outcomes of the four groups of sevoflurane or desflurane anesthesia with or without M-Entropy guidance.

	SEVO without M-Entropy *n* = 20		SEVO with M-Entropy *n* = 20		DES without M-Entropy *n* = 20		DES with M-Entropy *n* = 20		*p*
Time to spontaneous eye opening, s	409	239, 570 (185, 982)	463	330, 564 (149, 814)	371	254, 488 (125, 778)	218	154, 333 (121, 495)	0.0012
Time to obeying commands, s	532	353, 663 (235, 1158)	488	442, 591 (210, 884)	400	318, 537 (180, 825)	310	203, 368 (130, 548)	0.0001
Time to tracheal extubation, s	575	398, 712 (255, 1066)	571	489, 671 (232, 1047)	449	362, 604 (205, 1144)	313	197, 385 (160, 617)	<0.0001
Time to leaving operating room, s	791	665, 936 (443, 1372)	851	738, 908 (548, 1400)	765	656, 849 (490, 1255)	569	459, 702 (410, 865)	0.0003
Emergence agitation	10	50.0	3	15.0	7	35.0	3	15.0	0.0370
Drowsiness after tracheal extubation	0	0	3	15.0	2	10.0	0	0	0.1591
Intraoperative awareness or recall	0	0	0	0	0	0	0	0	NA
Time percentage of RE > 60	23.0	9.5, 50.6 (1.9, 77.3)	26.4	17.1, 37.1 (5.0, 66.7)	10.5	7.2, 18.8 (3.2, 51.5)	12.5	7.4, 20.4 (2.5, 47.1)	0.0049
Time percentage of RE ranged 40–60	53.3	36.7, 71.8 (22.2, 90.7)	71.1	52.2, 80.0 (27.8, 92.5)	48.7	28.6, 67.2 (9.7, 89.5)	82.9	75.0, 88.0 (52.9, 95.0)	<0.0001
Time percentage of RE < 40	10.0	0, 30.2 (0, 55.6)	1.3	0, 5.6 (0, 14.3)	33.2	7.5, 61.7 (0, 87.1)	0	0, 9.9 (0, 19.2)	<0.0001
Average RE value	52	48, 64 (42, 70)	58	56, 61 (51, 68)	46	41, 54 (36, 62)	53	50, 55 (47, 61)	<0.0001
Time percentage of SE > 60	20.6	9.2, 42.9 (1.9, 77.3)	21.1	14.3, 32.6 (3.4, 61.1)	9.0	5.3, 16.8 (3.2, 51.5)	12.5	7.4, 16.7 (2.5, 41.2)	0.0101
Time percentage of SE ranged 40–60	49.1	39.6, 71.8 (22.2, 88.9)	77.8	57.6, 81.9 (33.3, 92.5)	51.3	25.0, 68.8 (9.7, 84.2)	78.5	74.4, 84.6 (53.8, 95.0)	<0.0001
Time percentage of SE < 40	10.7	0, 33.3 (0, 55.6)	1.3	0, 5.6 (0, 14.3)	34.9	11.1, 65.9 (0, 87.1)	4.0	0, 14.3 (0, 28.6)	<0.0001
Average SE value	50	46, 62 (41, 68)	56	54, 59 (49, 66)	45	38, 53 (35, 60)	50	48, 53 (45, 60)	0.0001
Average level of end-tidal SEVO, %	1.66	1.57, 1.76 (1.21, 2.16)	1.54	1.46, 1.83 (1.33, 2.29)	NA	NA	NA	NA	0.4091
Average level of end-tidal SEVO, aaMAC	0.78	0.73, 0.83 (0.57, 0.99)	0.73	0.69, 0.84 (0.63, 1.12)	NA	NA	NA	NA	0.4165
Average level of end-tidal DES, %	NA	NA	NA	NA	4.85	4.60, 5.23 (3.65, 6.83)	4.41	4.03, 5.09 (2.83, 5.95)	0.0531
Average level of end-tidal DES, aaMAC	NA	NA	NA	NA	0.73	0.67, 0.80 (0.51, 0.95)	0.64	0.59, 0.75 (0.42, 0.85)	0.0358

Values are counts with percent or median with interquartile range (minimum and maximum). Abbreviation: aaMAC, age-adjusted minimum alveolar concentration; DES, desflurane; NA, not applicable; RE, response entropy, SE: state entropy; SEVO, sevoflurane.

**Table 5:** Intergroup comparisons for study outcomes.

	SEVO/EG vs. SEVO/NEG		DES/NEG vs. SEVO/NEG		DES/EG vs. SEVO/NEG		DES/NEG vs. SEVO/EG		DES/EG vs. SEVO/EG		DES/EG vs. DES/NEG	
	MD or RD (99.2% CI)	*p*	MD or RD (99.2% CI)	*p*	MD or RD (99.2% CI)	*p*	MD or RD (99.2% CI)	*p*	MD or RD (99.2% CI)	*p*	MD or RD (99.2% CI)	*p*
Time to spontaneous eye opening, s	4 (−179, 186)	0.9532	−55 (−237, 126)	0.3966	−198 (−357, −39)	0.0014	−59 (−222, 104)	0.3145	−202 (−338, −66)	0.0002	−142 (−276, −8)	0.0052
Time to obeying commands, s	−34 (−214, 146)	0.5969	−109 (−299, 81)	0.1175	−245 (−412, −79)	0.0002	−74 (−234, 85)	0.1992	−211 (−337, −85)	<0.0001	−137 (−279, 6)	0.0106
Time to tracheal extubation, s	14 (−168, 196)	0.8302	−65 (−272, 141)	0.3810	−265 (−422, −107)	<0.0001	−79 (−279, 120)	0.2726	−279 (−425, −132)	<0.0001	−199 (−379, −19)	0.0037
Time to leaving operating room, s	47 (−144, 237)	0.4946	−30 (−223, 163)	0.6681	−220 (−383, −57)	0.0005	−77 (−271, 118)	0.2762	−267 (−432, −102)	<0.0001	−190 (−358, −23)	0.0029
Emergence agitation, %	−35.0 (−71.4, 1.4)	0.0181	−15.0 (−56.0, 26.0)	0.3373	−35.0 (−71.4, 1.4)	0.0181	20.0 (−15.3, 55.3)	0.1441	0 (−30.0, 30.0)	>0.9999	−20.0 (−55.3, 15.3)	0.1441
Drowsiness after tracheal extubation, %	15.0 (−6.2, 36.2)	0.2308	10.0 (−7.8, 27.8)	0.4872	NA	NA	−5.0 (−32.7, 22.7)	>0.9999	−15.0 (−36.2, 6.2)	0.2308	−10.0 (−27.8, 7.8)	0.4872
Intraoperative awareness or recall	0 (0, 0)	NA	0 (0, 0)	NA	0 (0, 0)	NA	0 (0, 0)	NA	0 (0, 0)	NA	0 (0, 0)	NA
Time percentage of RE > 60	−2.7 (−21.9, 16.5)	0.6958	−17.0 (−35.0, 0.9)	0.0113	−16.4 (−34.1, 1.3)	0.0130	−14.3 (−27.4, −1.3)	0.0039	−13.7 (−26.5, 1.0)	0.0047	0.6 (−9.2, 10.4)	0.8630
Time percentage of RE ranged 40–60	14.5 (−2.4, 31.4)	0.0210	−4.9 (−24.2, 14.4)	0.4832	27.0 (12.4, 41.6)	<0.0001	−19.4 (−37.7, −1.1)	0.0052	12.5 (−0.6, 25.6)	0.0113	31.9 (15.5, 48.3)	<0.0001
Time percentage of RE < 40	−11.8 (−23.6, 0.1)	0.0083	21.9 (0.6, 43.3)	0.0066	−10.6 (−22.7, 1.6)	0.0186	33.7 (14.5, 52.8)	<0.0001	1.2 (−3.6, 6.0)	0.4890	−32.5 (−51.8, −13.2)	<0.0001
Average RE value	4 (−3, 10)	0.1121	−8 (−15, −0.2)	0.0067	−2 (−8, 4)	0.3564	−11 (−17, −5)	<0.0001	−6 (−9, −2)	<0.0001	6 (−0.04, 11)	0.0084
Time percentage of SE > 60	−3.9 (−21.8, 14.1)	0.5499	−15.6 (−32.6, 1.3)	0.0137	−14.6 (−31.1, 1.9)	0.0173	−11.8 (−24.2, 0.7)	0.0119	−10.7 (−22.5, 1.0)	0.0146	1.1 (−8.4, 10.5)	0.7558
Time percentage of SE ranged 40–60	17.7 (1.4, 34.0)	0.0042	−6.1 (−25.3, 13.1)	0.3791	24.0 (9.5, 38.5)	<0.0001	−23.8 (−41.6, −6.0)	0.0006	6.3 (−6.0, 18.6)	0.1583	30.1 (13.8, 46.5)	<0.0001
Time percentage of SE < 40	−13.8 (−27.3, −0.4)	0.0046	21.8 (−0.5, 44.0)	0.0094	−9.5 (−23.6, 4.7)	0.0656	41.2 (19.5, 63.0)	<0.0001	4.4 (−2.2, 10.9)	0.0669	−31.2 (−51.2, −11.2)	0.0002
Average SE value	3 (−3, 10)	0.1253	−7 (−15, −0.03)	0.0078	−2 (−8, 4)	0.3404	−11 (−16, −5)	<0.0001	−5 (−9, −2)	0.0001	5 (−0.3, 11)	0.0112
Average level of end-tidal SEVO, %	0.001 (−0.23, 0.23)	0.9904	NA	NA	NA	NA	NA	NA	NA	NA	NA	NA
Average level of end-tidal SEVO, aaMAC	−0.001 (−0.11, 0.11)	0.9895	NA	NA	NA	NA	NA	NA	NA	NA	NA	NA
Average level of end-tidal DES, %	NA	NA	NA	NA	NA	NA	NA	NA	NA	NA	−0.46 (−1.13, 0.22)	0.0673
Average level of end-tidal DES, aaMAC	NA	NA	NA	NA	NA	NA	NA	NA	NA	NA	−0.08 (−0.18, 0.02)	0.0231

Abbreviation: aaMAC, age-adjusted minimum alveolar concentration; CI, confidence interval; DES, desflurane; EG, M-Entropy guidance; MD, mean difference; NA, not applicable; NEG, no M-Entropy guidance; RD, risk difference; RE, response entropy, SE: state entropy; SEVO, sevoflurane.

## Data Availability

The data presented in this study are available on request from the corresponding author. The data are not publicly available due to the regulations of the Institutional Review Board.

## Supplementary Materials

The following supporting information can be downloaded at: https://www.mdpi.com/article/10.3390/jcm11010162/s1, Table S1: Intraoperative hemodynamic parameters.
