# Supplementary material for: Recovery Profiles of Sevoflurane and Desflurane with or without M-Entropy Guidance in Obese Patients: A Randomized Controlled Trial
Source: J Clin Med. 2021 Dec 29;11(1):162. doi: 10.3390/jcm11010162 (PMC8745589; doi:10.3390/jcm11010162)
Supplement: Supplementary file 1 [file jcm-11-00162-s001.zip › jcm-1479042-supplementary.pdf]

Table S1. Intraoperative hemodynamic parameters.

	SEVO without M-Entropy $n = 20$		SEVO with M-Entropy $n = 20$		DES without M-Entropy $n = 20$		DES with M-Entropy $n = 20$		p
**Before induction**									
Heart rate, beat·min^{-1}	82	69, 96 (58, 114)	78	71, 84 (63, 108)	74	68, 86 (59, 126)	81	69, 90 (56, 116)	0.7824
Mean arterial pressure, mm Hg	110	102, 114 (88, 140)	101	91, 121 (79, 140)	112	98, 121 (74, 147)	100	95, 112 (92, 128)	0.3185
Body temperature, °C	36.2	35.9, 36.5 (35.4, 36.9)	36.2	35.9, 36.4 (34.2, 36.9)	36.4	36.1, 36.4 (35.5, 36.9)	36.3	36.0, 36.5 (32.6, 36.8)	0.7700
SpO_2, %	97	96, 98 (94, 100)	98	97, 98 (93, 100)	97	96, 98 (94, 100)	98	97, 98 (95, 100)	0.8579
**5 min after induction**									
Heart rate, beat·min^{-1}	95	81, 101 (65, 128)	94	82, 100 (71, 157)	92	83, 101 (59, 130)	96	84, 104 (72, 112)	0.9663
Mean arterial pressure, mm Hg	83	72, 93 (62, 111)	79	71, 95 (55, 113)	91	77, 98 (46, 128)	86	80, 93 (72, 105)	0.4796
Body temperature, °C	36.1	35.7, 36.3 (32.9, 36.5)	36.0	35.7, 36.2 (34.2, 36.7)	36.1	35.6, 36.4 (34.6, 36.9)	36.2	35.8, 36.5 (32.2, 36.7)	0.6657
SpO_2, %	99	98, 99 (97, 100)	99	98, 99 (90, 100)	99	98, 100 (95, 100)	99	98, 99 (96, 100)	0.6549
**5 min after start of**									
Heart rate, beat·min^{-1}	93	79, 97 (66, 117)	93	81, 99 (47, 133)	90	78, 101 (61, 119)	86	78, 97 (67, 112)	0.8170
Mean arterial pressure, mm Hg	79	62, 101 (41, 137)	90	73, 102 (65, 125)	83	62, 113 (45, 124)	87	75, 101 (49, 149)	0.7186
Body temperature, °C	36.2	35.8, 36.3 (32.9, 36.7)	35.9	35.7, 36.2 (34.8, 36.9)	36.2	36.0, 36.5 (35.2, 36.7)	36.3	36.1, 36.5 (35.7, 37.0)	0.0381
SpO_2, %	99	97, 100 (94, 100)	98	97, 99 (93, 100)	98	97, 99 (91, 100)	99	97, 99 (97, 100)	0.7239
**5 min after end of**									
Heart rate, beat·min^{-1}	91	82, 98 (64, 115)	88	80, 97 (60, 141)	89	80, 97 (67, 119)	90	77, 98 (63, 105)	0.9519
Mean arterial pressure, mm Hg	99	88, 112 (79, 126)	93	85, 105 (67, 117)	100	93, 111 (81, 121)	100	92, 108 (69, 118)	0.3739
Body temperature, °C	36.3	36.1, 36.5 (32.6, 36.9)	36.2	35.8, 36.6 (35.0, 36.9)	36.4	36.3, 36.5 (36.1, 37.0)	36.5	36.2, 36.7 (35.2, 37.3)	0.2132
SpO_2, %	99	98, 99 (91, 100)	98	97, 99 (94, 100)	98	97, 99 (95, 100)	98	96, 99 (95, 100)	0.3904
**Cessation of volatile anesthetics**									
Heart rate, beat·min^{-1}	86	78, 101 (66, 114)	94	82, 102 (63, 148)	94	85, 101 (65, 118)	89	79, 96 (67, 108)	0.5568
Mean arterial pressure, mm Hg	104	96, 119 (69, 131)	110	100, 117 (74, 132)	118	108, 121 (70, 132)	109	99, 115 (71, 131)	0.2036
Body temperature, °C	36.4	35.9, 36.7 (32.9, 36.9)	36.2	36.1, 36.7 (35.2, 37.0)	36.4	36.2, 36.6 (35.8, 37.0)	36.4	36.2, 36.8 (35.5, 37.2)	0.6126
SpO_2, %	99	98, 100 (95, 100)	99	98, 100 (94, 100)	98	97, 100 (86, 100)	99	97, 100 (96, 100)	0.4999
**5 min after tracheal extubation**									
Heart rate, beat·min^{-1}	95	86, 109 (68, 131)	91	84, 99 (75, 128)	96	86, 99 (71, 115)	95	88, 102 (74, 116)	0.6966
Mean arterial pressure, mm Hg	106	100, 114 (85, 139)	105	96, 110 (82, 136)	108	104, 118 (84, 138)	102	95, 117 (66, 154)	0.2973
Body temperature, °C	36.4	36.2, 36.7 (35.6, 36.9)	36.5	36.2, 36.7 (36.0, 37.0)	36.5	36.2, 36.8 (35.8, 37.2)	36.7	36.4, 36.8 (36.1, 37.2)	0.2758
SpO_2, %	98	96, 99 (92, 100)	98	97, 100 (92, 100)	98	96, 99 (94, 100)	99	97, 100 (94, 100)	0.3338

Values are median with interquartile range (minimum and maximum). Abbreviations: DES, desflurane; SEVO, sevoflurane; SpO_2, peripheral capillary oxygen saturation.
